# Targeting Mitochondrial Protein Expression as a Future Approach for Cancer Therapy

**DOI:** 10.3389/fonc.2021.797265

**Published:** 2021-11-23

**Authors:** Daniela Criscuolo, Rosario Avolio, Danilo Swann Matassa, Franca Esposito

**Affiliations:** Department of Molecular Medicine and Medical Biotechnology, University of Naples “Federico II”, Naples, Italy

**Keywords:** mitochondrial translation, protein synthesis, inter-organelle coordinated translation regulation, mitochondrial protein import, mitochondrial protein quality control (mtPQC)

## Abstract

Extensive metabolic remodeling is a fundamental feature of cancer cells. Although early reports attributed such remodeling to a loss of mitochondrial functions, it is now clear that mitochondria play central roles in cancer development and progression, from energy production to synthesis of macromolecules, from redox modulation to regulation of cell death. Biosynthetic pathways are also heavily affected by the metabolic rewiring, with protein synthesis dysregulation at the hearth of cellular transformation. Accumulating evidence in multiple organisms shows that the metabolic functions of mitochondria are tightly connected to protein synthesis, being assembly and activity of respiratory complexes highly dependent on *de novo* synthesis of their components. In turn, protein synthesis within the organelle is tightly connected with the cytosolic process. This implies an entire network of interactions and fine-tuned regulations that build up a completely under-estimated level of complexity. We are now only preliminarily beginning to reconstitute such regulatory level in human cells, and to perceive its role in diseases. Indeed, disruption or alterations of these connections trigger conditions of proteotoxic and energetic stress that could be potentially exploited for therapeutic purposes. In this review, we summarize the available literature on the coordinated regulation of mitochondrial and cytosolic mRNA translation, and their effects on the integrity of the mitochondrial proteome and functions. Finally, we highlight the potential held by this topic for future research directions and for the development of innovative therapeutic approaches.

## 1 Introduction

Mitochondria are cellular organelles with a double-membrane structure that perform several crucial functions for the homeostasis of eukaryotic cells. Their main role is to generate chemical energy through the oxidative phosphorylation (OXPHOS) system, which is composed of five multi-subunit respiratory complexes associated to the inner mitochondrial membrane (IMM). Additionally, they work as biosynthetic hubs for the synthesis of amino acids, nucleotides, lipid heme and iron-sulphur clusters (1, 2). Moreover, mitochondria control the redox homeostasis and regulate cell death pathways (3, 4).

Mammalian mitochondria originate from the endocytosis of a bacterial ancestor by a pre-eukaryotic cell (5). Although mitochondria still maintain their own genome, during evolution most of the original endosymbiont genes were lost or transferred to the nuclear genome of the host cell (6). The mitochondrial DNA (mtDNA) is found in multiple copies in the mitochondrial matrix and, in humans, consist of approximately 16,000 base pairs encoding 13 polypeptides, all of which are key subunits of the OXPHOS complexes, 2 mitochondrial ribosomal RNA (mt-rRNA) and 22 transfer RNA (mt-tRNAs), that allow the translation of the 13 mitochondrial protein-coding RNAs. Indeed, translation of these mitochondrial messenger RNAs (mt-mRNAs) requires a dedicated translation apparatus, which is located in the mitochondrial matrix and includes mt-rRNAs, mt-tRNAs, nuclear-encoded translation factors and organelle-specific ribosomes. However, most mitochondria-resident proteins, including many subunits of the OXPHOS complexes, are encoded by the nuclear genome, synthesized by cytoplasmic ribosomes and then imported into the mitochondria (7). Therefore, accurate assembly of respiratory complexes requires a tight coordination between cytosolic and mitochondrial translation and efficient protein quality control (PQC) mechanisms to monitor protein import and turnover (8, 9).

Despite early theories on the metabolic characteristics of cancer cells postulated a loss of mitochondrial functions as a key feature of cellular transformation, it is now evident that this feature is often crucial for tumor development and progression (10). Moreover, several studies have shown that different cancer cells predominantly use mitochondrial respiration to satisfy their bioenergetic and biosynthetic demands, especially when moving towards a metastatic or chemoresistant phenotype (11–13). Accordingly, upregulation of the mitochondrial translational machinery has been reported to support the energy needs of cancer cells favoring tumor progression. Therapeutic approaches that interfere with mitochondrial translation, directly, by targeting mitoribosomes, or indirectly, by altering mitochondrial PQC systems, have recently attract great attention as anticancer strategies.

Here, we review the main mechanisms affecting mitochondrial protein homeostasis. First, we provide an overview of mitochondrial translation, and we focus on how it is strictly interconnected to the cytosolic apparatus. Then, we describe the importance of mitochondrial protein quality control systems in coordinating these translational programs, and present the case of the molecular chaperone TRAP1, likely first example of a protein with dual localization that participate in the regulation of proteostasis on both sides of the mitochondrial membranes. Finally, we provide some hints about dysfunctions of mitochondrial protein homeostasis and cancer development, highlighting the most relevant therapeutic approaches proposed so far in the field.

## 2 Regulation of Mitochondrial Gene Expression

Mitochondria have their own genome and translational machinery that allow synthesis and assembly of OXPHOS complexes, which are in turn responsible for the generation of most of the cellular energy. The mtDNA is compacted with an array of proteins in a structure called “nucleoid” that resembles the bacterial one. The protein components of the nucleoid are transcription and replication factors such as the mitochondrial transcription factor A (TFAM), mitochondrial single-strand binding protein (mtSSB), POLG, and mtRNA polymerase (POLRMT) (14). Other factors seem not to bind directly mtDNA, but are rather peripheral nucleoid proteins involved in scaffolding, helping translation and interaction with cellular signaling components (14). Among these ADAT3 (ATPase AAA domain-containing protein 3), PHB1 (Prohibitin 1), PHB2 and M19/MNF1 (mitochondrial nucleoid factor 1). The core nucleoid component POLRMT, an RNA polymerase structurally similar to the T3 and T7 bacteriophages one, in association with TFAM, is instead responsible for the transcription process. Notably, it has been recently described the first-in-class specific inhibitor of mitochondrial transcription that target the human POLRMT. This compound (IMT1) has shown relevant anti-tumor effects in mouse xenograft, with no significant toxicity in normal tissues (15). These findings represent a promising novel weapon in the fight for cancer treatment, but also a useful tool to study the role of mtDNA expression in physiology and disease.

However, to the current knowledge, mitochondrial gene expression is predominantly regulated at post-transcriptional level through the modulation of mRNA maturation and stability (16). Transcription of the mitochondrial genome by the RNA polymerase generates long polycistronic precursors containing mRNA and rRNA coding sequences flanked by tRNAs. Mitochondrial RNAs are processed by two endonucleases, RNase P and RNase Z (ELAC2), which cleave the 5’- and 3’-ends respectively, excising the tRNAs and releasing the rRNAs and mRNAs, a process known as tRNA punctuation (17). Subsequently, all mt-mRNAs, except ND6, are polyadenylated at 3’ termini by the mitochondrial polyA polymerase (mtPAP) (18). The mt-mRNA polyadenylation creates a functional stop codon, as 7 of 13 transcripts have incomplete stop codons in their coding sequence (19). Moreover, polyadenylation regulates the half-life of specific subset of mRNAs, increasing the stability of some transcripts and decreasing that of others by targeting them for degradation (20). Proteins that regulate mt-mRNA maturation and degradation determine which subsets of mitochondrial transcripts have to be translated, by affecting the availability of functional transcripts that can be engaged by the mitoribosomes.

LRPPRC (leucine-rich pentatricopeptide repeat containing), and Fas-activated serine/threonine kinase (FASTK) are protein families playing a major role in mt-mRNA stability and translation and whose dysregulation is related to diverse pathological processes, including cancer. LRPPRC, in complex with SLIRP (SRA stem loop-interacting RNA-binding protein), behaves as a mRNA chaperone, by preventing the formation of secondary structures, and affects the stability of mitochondrial transcriptome (21, 22). In particular, LRPPRC-SLIRP complex promotes mt-mRNA stability, by preventing their degradation, and polyadenylation, by simulating mtPAP activity (22). Moreover, LRPPRC-SLIRP complex has also been shown to stabilize a pool of non-translating transcripts that are not engaged with mitoribosomes (23). It is clear that LRPPRC-SLIRP complex is necessary for coordinated mitochondrial translation as its loss causes dysregulations, increasing translation of some transcripts and inhibiting translation of others (23, 24). Interestingly, human LRPPRC–SLIRP complex preferentially binds the human mt-Cyb RNA, whereas the mouse complex preferentially recognized the mouse corresponding transcript (21), and therefore that preferred locations of LRPPRC-binding sites within mitochondrial RNAs differ from mouse to humans. This testifies the importance of these proteins in dictating the local RNA structures that are critical in the lifecycles of mitochondrial RNAs. Several studies have shown that LRPPRC is upregulated in different cancer tissues and cell lines, including prostate, gastric, lung and colon cancer (25). Moreover, LRPPRC has been proposed as prognostic biomarker for gastric cancer. Indeed, a higher LRPPRC expression was found in cancer tissues compared to paired noncancerous regions and in patients with a poor survival rate (26).

FASTK family proteins are particularly expressed in the mitochondrial matrix, where they post-transcriptionally regulate the expression of different mitochondrial transcripts (27). For instance, FASTK interacts with the ND6 mRNA to prevent its degradation, by ensuring correct biogenesis of the complex I, whereas FASTKD1 negatively regulates complex I activity by destabilizing the ND3 mRNA (28, 29). Recently, a pan-cancer analysis showed that FASTK genes are frequently mutated in different cancer types highlighting the potential role of FASTK family members as therapeutic targets. In particular, gene amplification was found for FASTK and FASTKD3 in ovarian and lung cancers, respectively, while increased mRNA levels of all FASTK members were found in esophageal, stomach, liver and lung cancers (30).

## 3 Overview of the Mitochondrial mRNA Translation Process

A detailed discussion of the complex mechanisms involved in mitochondrial translation is beyond the scope of this article. For a comprehensive view of this topic, we recommend further reading [e.g (31)]. Hereafter, we provide a brief overview of the mitochondrial translation process, as well as of synthesis and import of the nuclear-encoded, mitochondria-destined proteins, with the aim to highlight how recent advances on this topic could open new scenarios on the mechanisms involved in metabolic remodeling in diseases, providing solid bases for future therapeutic approaches.

Translation of mt-mRNAs occurs on specialized ribosome resident in the mitochondrial matrix: the mitoribosomes. The mammalian 55S mitoribosomes are macromolecular complexes composed of two subunits, the large 39S subunit (LSU) and the small 28S subunit (SSU). The 39S subunit contains 16S mt-rRNA and 52 mitoribosomal proteins (MRPs), whereas the 28S subunits is composed of 12S mt-rRNA and 30 MRPs (32). Mitochondrial rRNAs are exclusively encoded by mtDNA, whereas MRPs are all encoded by the nuclear genome, translated in the cytosol and then imported into the mitochondrial matrix to be assembled coordinately with mt-rRNAs to form functional ribosomes. Mitoribosomes assembly takes place in close proximity to mtDNA, probably in mitochondrial RNA granules or mitochondriolus, membraneless structures containing MRPs, mitoribosome assembly factors and rRNA modifying enzymes required for post-transcriptional processing of mt-RNAs (33, 34).

**Figure 1:**
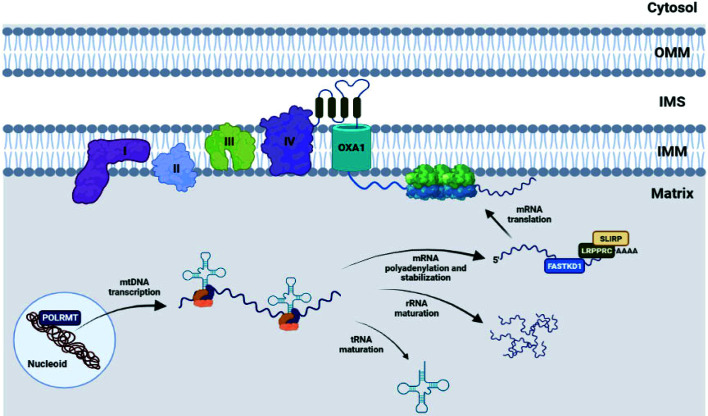
The mtDNA is organized in structures called “nucleoids”, in which both core and peripheral proteins contribute to organization, stability and communication of the mtDNA with additional factors. Among the nucleoid components, POLRMT plays a key role, being responsible for the transcription process. Subsequently, the original polycistronic transcript is subject to extensive maturation, yielding mitochondrial tRNAs, rRNAs and mRNAs. The latter encode 13 polypeptides, all members of the respiratory chain, can be stabilized and regulated by RNA-binding proteins such as LRPPRC, SLIRP FASTKD1, and finally translated by inner membrane-tethered mitoribosomes, to be cotranslationally assembled into the OXPHOS complexes. I, II, III, IV: respiratory complexes I-IV.

Although mitoribosomes are evolutionarily derived from bacterial ribosomes, they have strongly diverged from them in terms of composition, function, and structure, by acquiring mitochondrial-specific proteins, and exhibiting differences in the number and total amount of the rRNAs. These structural changes have been accompanied by a strong functional specialization, considering that mammalian mitoribosomes exclusively synthesize membrane proteins, represented by components of the respiratory complexes, which functionally explains the acquired feature of mitochondrial ribosomes to be permanently attached to the IMM (32). The 13 proteins encoded by the mtDNA are indeed all subunits of respiratory chain complexes and, as such, are highly hydrophobic polypeptides predominantly associated with the IMM. To avoid unproductive protein aggregations, the mitochondrial translation products are cotranslationally inserted into the IMM (35). Accordingly, the mitoribosomes extensively interact with the IMM to facilitate the membrane insertion of nascent polypeptides. In particular, the LSU subunit MRPL45 anchors the mitoribosome to the IMM aligning the polypeptide exit tunnel with the insertase OXA1L, that mediates the co-translational insertion of newly synthetized proteins into the IMM (36, 37) (**Figure 1**).

Mitochondrial translation begins when a mt-mRNA is loaded onto the SSU, then a start codon is recognized by the initiator tRNA carrying a formylated methionine (fMet-tRNA^Met^) (38). Before mRNA loading, two mitochondrial initiation factors (mIFs), mIF2 and mIF3, assemble on the SSU. Initially, mtIF3 binds SSU to prevent the premature reassociation with LSU and avoid binding of fMet-tRNA^Met^ to the P-site in the absence of mRNA and mtIF2 (39). Subsequently, mtIF2:GTP binds the SSU and promotes the binding of fMet-tRNA^Met^ to the P site while avoiding the association of tRNAs to the ribosomal A site. Following correct codon-anticodon interaction between fMet-tRNA^Met^ and the start codon, LSU joins the SSU forming the monosome, mtIF2 hydrolizes GTP to GDP and the initiation factors are released from the ribosome resulting in the mature ribosome ready to enter the elongation phase (38).

Currently, how mitochondrial transcripts are loaded onto the mitoribosomes and how the start codon is selected is unclear, as human mt-mRNAs lack the Shine–Dalgarno or the Kozak sequences, the most common cis-regulatory elements located at the 5’ UTRs of the prokaryotic and eukaryotic mRNAs respectively, that help to recruit mRNA to the ribosome and to recognize the start codon during translation initiation (40).

During elongation, selected amino acids are sequentially added to the nascent polypeptide. Aminoacyl-tRNAs are delivered to the A-site of mitoribosomes by the mitochondrial elongation factor EFTu (mtEFTu) bound to GTP. Upon correct codon-anticodon interaction, GTP is hydrolyzed to GDP and mtEFTu : GDP is released from the complex. The recycling of mtEFTu needs the elongation factor Ts (mtEFTs), that exchange GDP for GTP on mtEFTu, allowing it to bind and deliver the next aminoacylated tRNA (41). After the release of mtEF-Tu, the peptidyl transferase center in the LSU catalyzes the formation of the peptide bond between the nascent peptide chain of peptidyl-tRNA in the P site and the new amino acids carried by the aminoacyl-tRNA present in the A-site leaving a deacetylated tRNA in the P-site and one residue longer peptidyl-tRNA in the A-site (42). Subsequently, the mitochondrial elongating factor G1 (mtEFG1) catalyzes the GTP hydrolysis-dependent translocation of the mitoribosome, moving the deacylated tRNA from the P to the E-site and the peptidyl-tRNA from the A-site to the P-site, hence a new codon is exposed in the A-site and the cycle can start again (43).

The elongation cycle is reiterated until the polypeptide chain is completed and a stop codon reaches the ribosomal A-site. The stop codon association is recognized by the mitochondrial release factor 1a (mtRF1a) that mediates the hydrolysis of the ester bond between the last tRNA and the completed polypeptide, resulting in the release of the newly formed protein (44). Subsequently, two mitochondrial ribosome recycling factors, mtRRF1 and mtRRF2 (also known as mtEFG2), promote the dissociation of the mitoribosomal subunits and the release of mt-mRNA and deacylated mt-tRNA (45).

Aberrant expression of mitoribosomal proteins has been associated with several types of cancer in recent years (46). In breast cancer, analysis of genome-wide transcriptional profiling data and subsequent validation by immunohistochemistry, highlighted a significant enrichment in mitoribosomal proteins among the genes upregulated in the tumor tissue compared to the adjacent stroma. This suggests a tissue organization comprising highly oxidative epithelial breast cancer cells rich in mitochondria (and mitoribosomes), and a surrounding glycolytic stroma (47). On the other hand, lactate-mediated suppression of MRPL13 expression in hepatoma cells seems to promote hepatoma cell invasiveness and hepatocellular carcinoma development (48), highlighting the importance of the metabolic context in the contribution of mitochondrial protein synthesis to pathogenesis of human cancers.

## 4 Organelle-Localized Translation

In line with the endosymbiotic theory, mitochondria originate from a respiring proteobacterium, whose genome has been transferred during evolution into the nucleus of the eukaryotic host cell. Consequently, the vast majority of mitochondria-resident proteins are encoded by the nuclear genome, synthesized by the cytosolic translational machinery and imported into the mitochondria. The nuclear-encoded mitochondrial proteins are synthesized by cytosolic ribosomes as precursors bearing specific targeting signals that direct them to different mitochondrial sub-compartments, such as the N-terminal presequence required for a localization to the IMM or the matrix (49).

According to the classical view, once their synthesis is complete, preproteins are delivered on the mitochondrial surface in an unfolded state by molecular chaperones and then are imported *via* translocases of the outer mitochondrial membrane (OMM) and IMM (TOM/TIM complexes) (50, 51). However, experimental evidence suggest that mitochondria-destined proteins may be synthesized by cytosolic ribosomes localized near the OMM and co-translationally imported into the mitochondria (52, 53).

Already back in the 1970s, electron microscopy analysis found that cytoplasmic ribosomes can be localized near the OMM (54). Moreover, many microarray and RNA-seq analyses of biochemically fractionated mitochondria highlight the presence of nuclear-encoded mRNAs that are co-purified with mitochondria, and fluorescent microscopy analyses confirm these observations (55–58). Importantly, it has been shown that active translation is a key part of the localization process, as disassembly of polysomes by EDTA or puromycin treatment reduces the association between mRNAs and mitochondria (55, 59). In addition, ribosome profiling analyses performed on fractions of ribosomes isolated in the proximity of mitochondria confirmed that many IMM protein coding transcripts are co-translationally targeted to mitochondria (58). Delivery of mRNAs to the mitochondrial surface requires cis-acting signals, present in the transcripts or in the encoded polypeptide, and proteins that recognize these signals. Both 3′ UTR and coding regions, primarily through mitochondrial targeting sequences (MTSs), contribute to mitochondrial localization of transcripts (60, 61). In yeast, two classes of mRNAs that are translated near the mitochondria have been identified: class I mRNAs, bearing a binding motif in the 3’UTR recognized by the RNA-binding protein Puf3, and class II mRNAs, that are localized to mitochondria in a Puf3-independent manner (57). Both mRNA groups, independently transported on the mitochondrial surface, participate in the assembly of respiratory complexes: class I mRNAs encode assembly factors, whereas class II mRNAs encode structural proteins, indicating that differential regulation of mRNA localization near mitochondria is a potential mechanism to post-transcriptionally coordinate the construction of OXPHOS complexes (57). The TOM complex also participates to mRNA localization in both yeast and mammalian cells through interaction of protein receptor Tom20 with the MTS of the nascent polypeptide as it is translated (59, 62) instead, the outer-membrane protein OM14 is a mitochondrial receptor for the ribosome nascent-chain-associated complex (NAC), which interacts with both cytosolic translating ribosomes and nascent polypeptides as they emerged from exit tunnel (63). In *Drosophila* ovaries, the AKAP protein MDI, in complex with the translation stimulator La-related protein (Larp), promotes site-specific translation on the OMM of mRNAs encoding for mtDNA replication factors, mitochondrial ribosomal proteins, and electron-transport chain subunits, which is crucial for mitochondrial biogenesis during oogenesis (64).

These findings support the idea of co-translational import of nuclear-encoded proteins into mitochondria (65). Thus, the localization of transcripts in proximity of mitochondria and the activity of RNA-binding proteins as trans-acting factors provide a tool for a post-transcriptional regulation of gene expression at both a temporal and spatial level, to control protein import and respiratory complex assembly (56, 57, 66).

Finally, interactions between mitochondria and mRNA/nascent-peptide complexes can be altered by the kinetics of protein synthesis, which leads to enhanced protein expression for these factors during respiratory conditions (67). In this context, the length of translation time plays an important role in mRNA localization to the mitochondria, and it has been shown that increased translation time due to a translation elongation stall caused by polyproline sequences is one way exploited by yeast cell to extend the “competent state” of a translating mRNA to be recruited to the mitochondrion; moreover other mechanisms that increase translation duration such as increased ORF length, the presence of rare codons within the transcript, and mRNA structures could likely play a similar role in mRNA localization (67).

## 5 Import of Nuclear-Encoded Proteins Into the Mitochondria: The Assembly of Respiratory Complexes

As stated above, mitochondrial proteome is composed mostly of nuclear-encoded proteins that need to be imported into the mitochondria. Two transport mechanisms have been described, the post-translational and the co-translational translocation. Post-translational protein import implies that unfolded polypeptides, synthesized by cytosolic ribosomes, are guided to receptors of the TOM complex (Tom20, Tom22 and Tom70) by chaperones of the Hsp70, Hsp40, and Hsp90 families (50). Precursors containing the MTS are recognized by protein receptor Tom20, whereas Tom70 recognizes internal signals of hydrophobic polypeptides (68). Alternatively, ribosomes translating mitochondrial proteins can localize in the proximity of mitochondria through the action of RNA-binding proteins, such as Puf3 or NAC complex assembled on nascent polypeptides, controlling the co-translational translocation of proteins into mitochondria (63). In both cases, once transported across the OMM, mitochondrial precursors interact with the TIM23 complex, which transports preproteins into the IMM or matrix, aided by presequence translocase-associated motor (PAM) (69, 70).

TIM23 complex is emerging as a relevant factor for respiratory complex biogenesis, not only by regulating the import of presequence-carrying subunits, but also by promoting the incorporation of these subunits into the complexes. Indeed, TIM21, a subunit of TIM23 complex, is associated with complex I and complex IV assembly intermediates, where it transports the nuclear-encoded subunits for integration with the subunits encoded by the mitochondria (71). Moreover, TIM21 couples the TIM23 translocase to the cytochrome bc1-cytochrome c oxidase supercomplexes of the respiratory chain *via* a direct interaction with UQCR6, a subunit of complex III, supporting the import of presequence proteins under membrane potential limiting conditions, which makes it crucial for the import of protein when the membrane potential is reduced (72). Of note, it has been recently shown that integrity of complex III is crucial for the biogenesis and maturation of complex I and IV (73). Interestingly, in plants the TIM23 isoforms were found to associate with complex I (NADH dehydrogenase), too (74).

As proof of the intimate connection between respiratory chain functions and protein import, the succinate dehydrogenase (complex II) subunit Sdh3, which constitutes, along with Sdh4, a membrane integral module required for the recruitment of the catalytic subunits Sdh1 and Sdh2 to the IMM, is a moonlighting protein that participate, in partnership with Tim18, to the formation of TIM22 [reviewed in (75)]. Of note, Tim28 is also a close homolog of Shd4.

Once imported into the matrix *via* TIM23 complex, the precursor proteins must undergo the cleavage of their N-terminal presequences by the mitochondrial processing peptidase (MPP), which is crucial for the following folding and for the functionality of their catalytic activity. MPP consists of two homologous subunits, Mas1 and Mas2, that in turn are highly homologous to UQCRC1 and UQCRC2 core subunit of the respiratory complex III. Amazingly, it has been found in plants that Mas1 and Mas2 replace the core proteins and the MPP-activity is exclusively integrated into the complex (76).

It is noteworthy that these processes impact directly on the assembly and activity of respiratory complexes. In cancer biology, the contribution of mitochondrial metabolism to disease development and progression has long been underrated. This is due to the original hypothesis that cancer is a result of mitochondrial insufficiency, that was at the basis of Otto Warburg’s formulation of the “aerobic glycolysis” model. Although the Warburg effect remains central to our understanding of cancer cell metabolic remodeling, we now know that mitochondria play fundamental roles in several neoplasms such as sarcomas, cervical cancer, and melanomas (77), or, alternatively, in specific growth stages of the same tumor (78). Moreover, OXPHOS has been proven important to sustain survival and proliferation of chemoresistant cells (79). For these reasons, although our understanding of these phenomena in cancer cells is still at the early stages, it is reasonable to hypothesize that cancer cells must be particularly sensitive to uncoupling of mitochondrial and cytosolic translation, and to disruption of all the quality control networks highly connected to the electron transport chain functionality.

## 6 Mitochondrial Protein Quality Control

The coordinated expression and assembly of respiratory chain subunits, encoded by nuclear and mitochondrial genomes, require different PQC systems involving molecular chaperones and proteases that ensure the efficient import of nuclear encoded proteins, the correct folding of both nuclear and mitochondrial encoded proteins and the degradation of misfolded proteins or unassembled subunits (80).

Mitochondrial PQC occurs at the cytosolic side of the OMM to survey the import of nuclear-encoded proteins, in the intermembrane space and in the matrix to control their state and turnover. In this way, PQC occurs early on nascent polypeptides, which can be efficiently folded, modified and targeted to cellular membranes to avoid mis-localization, or rapidly ubiquitinated and degraded to prevent the accumulation of protein aggregates (81, 82).

The relevance of this phenomenon is testified by the recent discovery of a novel pathway of mitochondria-mediated cell death named mitochondrial Precursor Over-accumulation Stress (mPOS), that is characterized by aberrant accumulation of mitochondrial precursors in the cytosol (83). This condition is induced not only by mutations of components of the protein import machinery, but also by malfunction of the inner membrane. In keeping with these data, there is also evidence that cytosolic proteins are stabilized and mitochondrial protein import is reduced by condition of mitochondrial dysfunction (84–86). In this view, the positive effects evidenced by inhibition of mTOR on progression of mitochondrial diseases (87) could be interpreted as a result of decreases protein synthesis.

A second layer of PQC is exerted within the two sides of mitochondrial inner membrane by two ATP-dependent proteolytic complexes: the m-AAA complex, which functions at the matrix side of the membrane; and the i-AAA complex, whose role resides in the intermembrane space. The first comprises two isoenzymatic forms, the homo-oligomeric AFG3L2 subunits, implicated in the processing of Cox1 and MT-ATP6 respiratory chain subunits, and the hetero-oligomeric AFG3L2 and SPG7 subunits, involved in the degradation of the EMRE subunit of the mitochondrial calcium uniporter complex (88). The i-AAA complex plays a fundamental role in mitochondrial dynamics. Its subunit YMEL1, together with OMA1, was shown to regulate the processing of OPA1, thus affecting the process of mitochondrial fusion. Moreover, non-assembled Cox4, NDUFB6 and ND1 respiratory chain subunits, and the TIM23 subunit Tim17A, were all shown to be proteolitically processed by YMEL1 (89, 90).

Given the central role played by PQC in shaping mitochondrial functions following stressful conditions, it is not surprising that mitochondrial proteases could be relevant to the pathophysiology of some cancers. Accordingly, a recent study demonstrated how YMEL1 can rewire mitochondrial proteome to sustain the growth of pancreatic ductal adenocarcinoma (PDAC). PDAC is a solid tumor able to reprogram glutamine metabolism to overcome hypoxic and nutrient-deprived environment through the stabilization of HIF1α. Together with HIF1α stabilization, analysis of PDAC patient biopsies revealed that depletion of YMEL1 substrates represent a further mechanism encountered to optimize mitochondria metabolism rewiring and tumor progression. Indeed, depletion of YMEL1 was able to reduce both the growth of cultured PDAC cells as well as tumor formation *in vivo*. Conversely, the same effect was not observed for hepatocellular carcinoma, with HepG2 and Huh7 cell lines showing no differences in spheroids formation following YMEL1 silencing. These data suggest that the proteolytic rewiring by YMEL1 could strongly depend on both the metabolic needs of each tumor and the tumor microenvironment (91).

PQC is carried out in the mitochondrial matrix by molecular chaperones systems and proteases of the AAA+ (ATPase associated with diverse cellular activities) family that maintain the correct protein folding and remove the unfolded or damaged proteins and unassembled OXPHOS subunits (92, 93). The AAA+ proteases of the mitochondrial matrix include CLPXP and LON. CLPXP is a complex constituted by two components, the serine protease ClpP and the chaperone ClpX, that recognizes and delivers the protein substrates to ClpP for degradation (94). Mitochondrial LON protease plays a central role in the PQC in the mitochondrial matrix by removing unfolded and oxidized proteins and promoting the folding of imported proteins through interaction with the chaperone mtHSP70 (95, 96).

In addition to the degradation of unfolded proteins in the matrix, CLPX and LON regulate mitochondrial protein synthesis, and thus the biogenesis of respiratory complexes (97, 98). The ClpXP complex regulates mitoribosome assembly through degradation of ERAL1, a putative 12S rRNA chaperone essential for SSU assembly, but whose removal is necessary to form a mature mitoribosome and for translation initiation (97, 99). Loss of ClpP or loss of ClpXP activity affects mitoribosome assembly and reduces mitochondrial translation, leading to respiratory chain dysfunction (97). LON influences mitochondrial gene expression by regulating the degradation of mitochondrial transcription factor A (TFAM), essential for mtDNA transcription initiation, MRPP3A, the RNase P subunit responsible for mtRNA processing, and FASTKD2, a factor involved in the mitoribosome biogenesis (98, 100–102). Depletion of LON in human cells reduces the levels of mtDNA, impairs mature mitoribosomes assembly and thus abolishes mitochondrial protein synthesis (100, 101).

As expected, both LON and ClpP proteases levels correlate with tumor development (103). Indeed, the RNA levels of these proteases are up-regulated in several cancers, particularly in prostate cancer (104). Indeed, LON and ClpP synergistically cooperate to promote cell growth and survival of prostate cancer cells, with patients showing a worst survival outcome when the levels of both proteases are concomitantly high. This is in agreement with a significantly marked reduction in prostate cancer cell growth and increased sensitivity to metabolic stress inducers following silencing of the two proteases (104). The mechanism behind the tumorigenic role of LON and ClpP involves the PQC exerted on the SHMT2 enzyme, whose inhibition leads to a significant reduction in cell growth with a more pronounced effect when the proteases are depleted (104).

Perturbations of mitochondrial proteostasis leads to the activation of the mitochondrial unfolded protein response (mtUPR), a retrograde signal direct to nucleus aimed at maintaining the mitochondrial proteome integrity (105). Primarly, mtUPR attempts to relieve stress by inducing the expression of chaperones and proteases that increase mitochondrial protein folding capacity (106). In addition, in order to decrease the mitochondrial folding load, mtUPR reduces protein import and decreases mitochondrial translation by impairing mt-RNA processing and inducing the degradation of mt-mRNAs and MRPs (90, 100). The activation of mtUPR is a compensatory response that can be use by cancer cells as a cytoprotective strategy supporting adaptation to unfavorable milieus (107). However, prolonged activation of this stress response pathway can result in cell death. Therefore, targeting factors that control the protein folding environment within mitochondria has been explored as anticancer strategy. In this context, the molecular chaperone TRAP1 (Tumor Necrosis Factor Receptor-Associated Protein-1), the mitochondrial paralog of the HSP90 family, is recognized as a relevant factor in the control of mitochondrial homeostasis (108). TRAP1 is a gene of monophyletic origin only present in Animalia and some Protista, mostly similar to a eukaryotic HtpG (a *E. coli* heat shock protein), with the addition of a N-terminal transit peptide sequence for the targeting to mitochondria, likely evolved at the base of the TRAP1 lineage. TRAP1 has therefore arisen from the ancestral eukaryotes, and was not derived from an endosymbiont of bacterial origin (109). TRAP1 is indeed the only mitochondrial member of the HSP90 family, with high homology with cytosolic HSP90 though, holding an ATPase domain and an HSP90-domain involved in client protein binding that share an overall 26% identity and 45% similarity with cytosolic HSP90 (110). TRAP1 protects the mitochondria integrity under oxidative stress by preventing the permeability transition pore opening, through binding with cyclophilin D, and acting as downstream effector of PINK1 (111, 112). Relevant for this protective function is also a regulation of mitochondrial metabolism through both direct and indirect interaction with the respiratory chain (113), with relevant effects on cancer progression and drug resistance, especially in ovarian cancer (114–116). Moreover, it has been shown that genetic silencing or pharmacological inhibition of TRAP1 in human cancer cells induces the hallmarks of mtUPR signaling, including accumulation of unfolded matrix proteins and upregulation of multiple chaperones and stress response transcription factors CHOP and C/EBPβ (117). In addition to its role in the regulation of protein folding within the mitochondria, TRAP1 contributes to maintain the mitochondrial proteostasis, also acting in the cytosol. Indeed, TRAP1 is localized to the outer face of endoplasmic reticulum, where it interacts with both the proteasome and the ribosomes to regulate co-translational degradation of mitochondria-destined proteins such as F1ATPase beta subunit and a mitochondrial isoform of Sorcin (118, 119).

## 7 Coordination of Mitochondrial and Cytosolic Translation

All mitochondrial-encoded proteins participate in the formation of respiratory complexes together with nuclear encoding ones. Due to their dual genetic origin, the biogenesis of OXPHOS complexes requires the coordinated regulation of the mitochondrial and cytoplasmic translational machineries. The OXPHOS system subunits synthesized by mitochondrial translational machinery are: ND1-6 and ND4L for complex I (NADH dehydrogenase); cytochrome *b* for complex III (cytochrome *c* reductase); COX1-3 for complex IV (cytochrome *c* oxidase); ATP6 and ATP8 for complex V (ATP synthase). Hence, all the respiratory chain complexes but complex II have a dual genetic origin.

Studies performed in yeast demonstrate that expression of dual-origin OXPHOS complexes is induced upon adaptation to respiratory growth through a rapid and synchronous translation regulation across compartments, whereas OXPHOS mRNAs are not coordinately induced. Indeed, while nuclear transcripts are rapidly induced in response to a nutrient shift, the mitochondrial ones are induced more slowly, most likely reflecting the absence of mitochondrial transcription factor responsive to environmental changes (120). Synchronized translation could therefore serve to maximize the efficiency of OXPHOS complex assembly, especially in a mutable metabolic context, but also to limit nonproductive or harmful off-target interactions.

Of note, how the cytoplasmic and mitochondrial translation are synchronized in human cells is currently unknown. Conversely, studies in the yeast *Saccharomyces cerevisiae* extensively described feedback loops that coordinate mitochondrial translation with the availability of nuclear-encoded subunits to optimize the assembly of respiratory chain complexes. In particular, mitochondrial gene expression is regulated by several nuclear-encoded translational activators located into the IMM and in contact with the mitoribosomes (121). Translational activators tune the translation rate of specific mt-mRNAs to the import of nuclear encoded OXPHOS subunits to allow mitochondria to synthesize only those subunits that can be assembled into complexes, and thus avoiding the accumulation of unassembled subunits (121).

The best understood regulatory feedback of mitochondrial translation is the one involved in the synthesis of COX1 during the assembly of complex IV. The translational activator Mss51 binds Cox1 mRNA to start its translation, and interacts with the newly synthesized COX1 polypeptide in a pre-complex temporary assembled with the assembly factors Coa1, Coa3, Cox14 and Shy1 (122–125). In this complex, Mss51 is unable to stimulate the translation of the Cox1 transcript until the COX1 protein associates with additional subunits, imported into the mitochondria during the complex assembly, releasing Mss51, which can initiate a new round of Cox1 synthesis (121).

As for mammalian mitochondria, the only translation activator identified is TACO1 (translational activator of cytochrome oxidase I), which is necessary for the efficient translation of COX1 (126). However, a regulatory feedback exerted by cytosolic translation products on mitochondrial translation has been identified in human cells as a mechanism for the complex IV assembly (127). During translation of the Cox1 mRNA, two inner membrane proteins, C12ORF62 (COX14) and MITRAC12 (COA3) interact with the nascent polypeptide, inducing translation elongation arrest (127). Stalled mitoribosomes resume Cox1 mRNA translation only when COX4, the first nuclear-encoded subunit incorporated into the complex, is imported (127). Therefore, human mitoribosomes display a translational plasticity to coordinate their protein synthesis rate with the influx of cytosolic OXPHOS subunits and the assembly of respiratory complexes. It will be interesting to investigate whether the translational plasticity regulates the assembly of others human OXPHOS complexes with dual genetic origin.

Remarkable for the aim of this issue, several works pursuing the inhibition of mitochondrial protein synthesis as a therapeutic strategy against different tumors have shown that it leads to a decoupling of cytosolic and mitochondrial translation and consequent reduction in cell proliferation and fitness (128–130), suggesting that cancer cells could be particularly sensitive to translation uncoupling. In this view, particularly relevant is the function of the lncRNA SAMMSON, aberrantly expressed in a large fraction of melanomas and hepatocellular carcinomas (131), that has been found to concertedly stimulate rRNA biogenesis and protein synthesis in both cytosol and mitochondria of tumor cells (132). As a result, SAMMSON confers a growth advantage to immortalized cells irrespective of their tissue of origin, and is able to transform immortalized cells of melanocytic origin, allowing tumor growth in nude mice. Of note, knockdown of SAMMSON decreases melanoma viability by impairing mitochondrial translation and inducing an mPOS-like response (133), and induces apoptosis even before any effect of its depletion on ribosome biogenesis and cytosolic protein synthesis could be observed (132), supporting the importance of the coordination between both mechanisms in tumor cells.

An additional “study case” in the context of human tumors is the mitochondrial chaperone TRAP1, that is involved in the control of respiration and mitochondrial PQC, but also in the regulation of mitochondrial translation. By using two complementary approaches, it has been found that one of the functions most heavily affected by inhibition of mitochondrial HSP90 activity is mitochondrial translation, with many ribosomal proteins found aggregated and misfolded following treatment with non-cytotoxic concentrations of the Hsp90-inhibitor Gamitrinib (134). In support of this, an immunoprecipitation mass spectrometry experiment has shown that the mitochondrial translation elongation factor mtEf-Tu and several components of the mitochondrial protein import complexes TOM/TIM are, among others, TRAP1 interactors (135). Accordingly, TOM40 was also found in an independent proteomic experiment in search of TRAP1 interactors in HeLa cells (136). These pieces of evidence preliminary suggest that a single chaperone with predominant mitochondrial localization but with described functions associated to protein synthesis and co-translational PQC in the cytosolic compartment could be involved in the regulated synthesis of mitochondrial proteins on both sides of the mitochondrial membranes.

**Figure 2:**
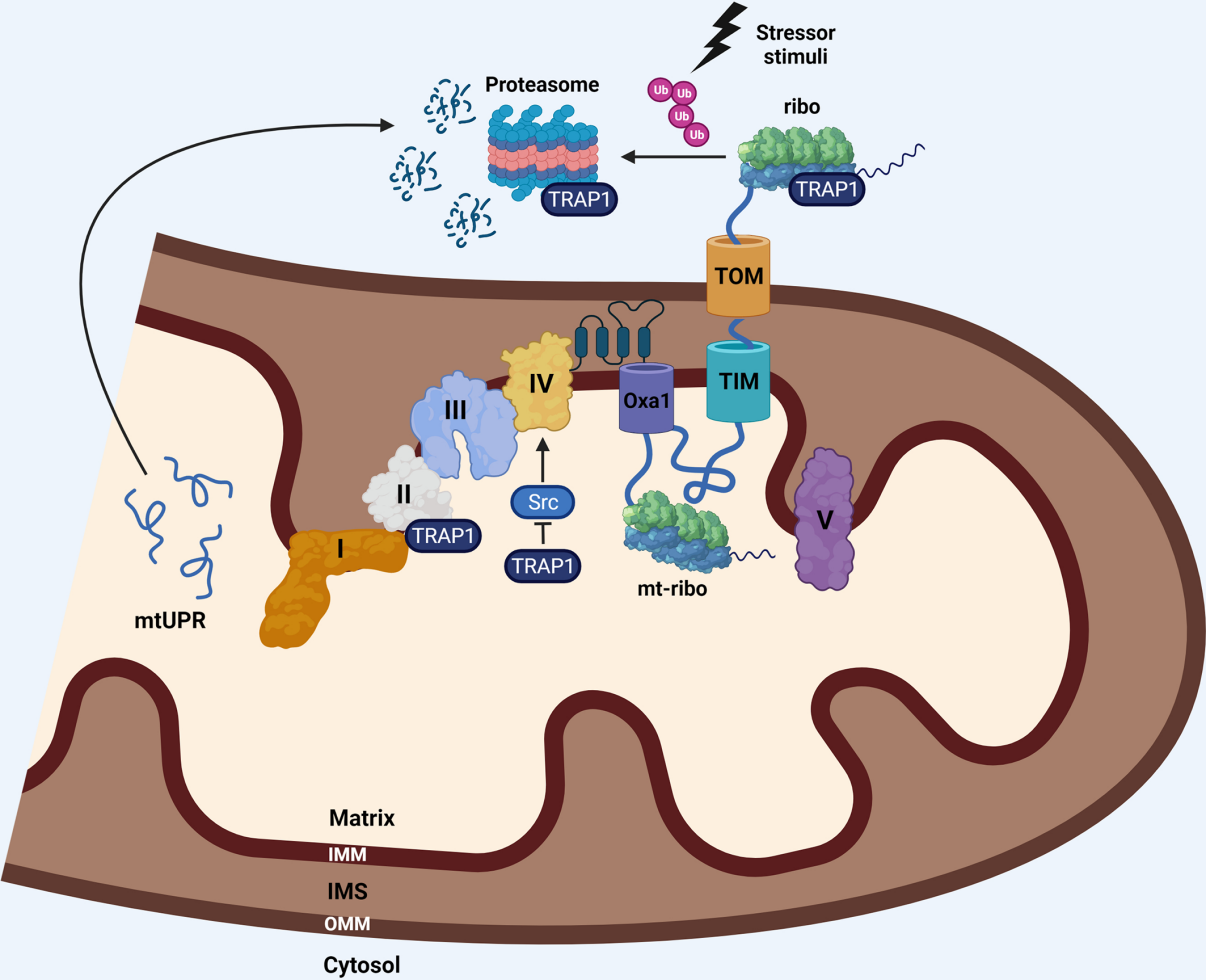
The nuclear-encoded mitochondrial proteins are synthesized by cytosolic ribosomes (ribo) that can be localized at the OMM, allowing co-translational import of nascent proteins into the organelle *via* the TOM/TIM complexes. Translating ribosomes can act as a platform for early PQC by ribosome-associated chaperones, including TRAP1, that, under stress conditions, prevents aberrant aggregation of proteins, directing them to co-translational ubiquitin-mediated proteasomal degradation. The imported proteins taking part to respiratory complexes are then assembled in supercomplexes along with the 13 components that are synthesized within the organelle by the mitochondrial ribosomes (mt-ribo). The co-translational insertion of these subunits into the IMM is mediated by OXA1, which is crucial for the assembly of functional respiratory complexes. The same molecular chaperone assisting PQC of mitochondrial proteins, TRAP1, is contemporary a regulator of respiration, through a direct binding to complex II, and an indirect regulation on complex IV, through the stabilization of the inactive form of c-Src, which is known to stimulate complex IV activity. Inhibition of TRAP1 leads to a mtUPR and related stress response. I, II, III, IV: respiratory complexes I-IV.

The coordination of processes that control the homeostasis of mitochondrial proteome, from cytosolic translation in proximity of mitochondria to PQC, protein import and mitochondrial translation, are schematically represented in **Figure 2**.

## 8 Mitochondrial Translation-Targeted Therapy in Human Cancers

Although glycolysis has long been considered the major metabolic pathway for ATP production in cancer cells, even under aerobic conditions, several studies have now shown that some types of cancer cells choose OXPHOS for their metabolic demands (137–139). Of note, the same TRAP1 protein with roles in mitochondrial PQC within/outside mitochondria, is also considered a bona fide OXPHOS regulator, through the direct binding to SDH and an activity control exerted on complex IV, which require the modulation of c-Src phosphorylation (113). Indeed, TRAP1 appears to be upregulated in predominantly glycolytic tumors, while it is downregulated in highly respiratory ones (13, 140). This suggests the existence of gene expression programs in which genes are clustered for the activation of metabolic plans to integrate energetic and biosynthetic demands with nutrient and oxygen availability. In this context, high mitochondrial translation may be required to support the bioenergetic needs of cancer cells. Additionally, emerging evidence suggests that the mitochondrial ribosomal proteins, beside their role in mitoribosomes assembly, also exhibit moonlight functions in the regulation of cell cycle progression and apoptosis signaling (141). Therefore, it is not surprising that altered expression of mitochondrial translational machinery components has been identified in different tumor types (46, 47, 142).

Inhibition of mitochondrial functions has been explored as therapeutic strategy for cancer treatment. Owing to their prokaryotic origin, mitoribosomes are susceptible to the inhibitory effect of some antibiotics commonly used to treat bacterial infections (143, 144). In addition, due to the tight coupling between translation across compartments, PQC mechanisms and assembly of respiratory complexes, as well as mechanism of protein import and mitochondrial protein homeostasis can be also promisingly targeted for therapeutic purposes in all the systems in which mitochondrial function is key for cell survival and/or proliferation.

The main compounds developed for their capacity to target at different levels homeostasis of the mitochondrial proteome are discussed below, and listed in **Table 1**.

**Table 1:** List of mitochondrial proteostasis targeting agents used as anticancer drugs in preclinical and clinical studies.

Drug	Mechanism of action	Tumor type	Type of study	Clinical Trial	References
Doxycycline	Inhibitor of mitochondrial translation	NSCLC, PC, CRC MBC NHLs	*In vitro* *In vivo* *In vivo*	NCT01847976 NCT02086591	(145)
COL-3	Inhibitor of mitochondrial translation	NSCLC, PC, CRC CNS KS	*In vitro* *In vivo* *In vivo*	NCT00004147 NCT00020683	(145, 146)
Tigecycline	Inhibitor of mitochondrial translation	DLBCLs NSCLC OC AML CML ALL HCC RCC	*In vitro* *In vitro* *In vitro/in vivo* +/- cisplatin *In vitro/in vivo* *In vitro/in vivo* +/venetoclax *In vivo* *In vivo* *In vitro/in vivo* +/- doxorubicin or vincristine *In vitro/in vivo* +/- cisplatin *In vitro/in vivo* +/- paclitaxel	NCT01332786 NCT02883036	(129, 147–153)
Actinonin	Inhibitor of mitochondrial peptide deformylase	BL BC, PC, LC, OC, BL,	*In vitro* *In vitro/in vivo*		(154, 155)
MitoBloCK-6	Erv1/ALR inhibitor	AML HCC	*In vitro* *In vitro*		(156, 157)
ONC201	ClpP activator	BC, EC PC ASC EC BC, EC	*In vitro* *In vitro +/- radiation* *In vivo* *In vivo* *In vivo*	NCT02250781 NCT03485729 NCT03394027	(158–160)
CDDO-Me	LONP1 inhibitor	PC, CRC, OC, NSCLC, BC ASC	*In vitro* *In vivo*	NCT00508807	(161)
Gamitrinib	Inhibitor of mitochondrial HSP90 and TRAP1 ATPase activity	BC, LC, PC ASC	*In vitro* *In vivo*	NCT04827810	(162)

ALL, Acute lymphoblastic leukemia; AML, Acute myeloid leukemia; ASC, advanced solid cancers; BC, Breast cancer; BL, Burkitt’s lymphoma; CNS, central nervous system tumors; CRC, Colorectal cancer; DLBCLs, Diffuse large B-cell lymphomas; EC, endometrial cancer; GB, glioblastoma; HCC, Hepatocellular carcinoma; KS, Kasposi’s sarcoma; LC, lung cancer; MBC, Metastatic breast cancer; NHLs, Non Hodgkin Lymphomas; NSCLC, Nonsmall cell lung cancer; OC, Ovarian cancer; PAD, Pancreatic adenocarcinoma; PC, Prostate cancer; RCC, Renal cell carcinoma.

### 8.1 Targeting Mitochondrial Translation Machinery

#### 8.1.1 Tetracycline Analogues

Tetracyclines are broad spectrum antibiotics discovered in the late 1940s as natural products of *Streptomyces aureofaciens* strain and currently used to treat a wide variety of bacterial infections (163). The bacteriostatic activity of tetracyclines depends on their capacity to inhibit the protein synthesis by preventing the interaction of aminoacyl-tRNAs with the A-site of the ribosome and thus the peptide elongation (164). Besides being antimicrobial agents, tetracycline analogues, such as doxycycline, COL-3 and tigecycline, have shown anti-tumor effects in several human cancers in both pre-clinical and clinical studies (129, 145, 165).

The anticancer effects of doxycycline and COL-3, semisynthetic and chemically modified tetracycline, respectively, were mainly related to their inhibitory effects on the expression and activation of matrix metalloproteases (166, 167). In fact, doxycycline and COL-3 exert antiangiogenic and antimetastatic activity in different cancer cell lines including leukemias, osteosarcoma, breast, colorectal and prostate cancer (166, 168–172).

Recently, it has been show that doxycyline and COL-3 antiproliferative and pro-apoptotic effects are related to the inhibition of mitochondrial protein synthesis with a decreased OXPHOS, resulting in a significant slowdown of proliferation rate (145, 173). Moreover, the reduction of IMM potential induced by tetracyclines yields oxidative stress, bringing the cancer cells closer to the apoptotic threshold (173).

Tigecycline, a third generation tetracycline, has been identified by a chemical screening as an effective drug in reducing the viability of leukemia cell lines (129). Anti-leukemic activity of tigecycline is due to the inhibition of mitochondrial translation which significantly reduces the OXPHOS capacity of cancer cells (129). Tigecyclin inhibition is selective for mitochondrial translation as it reduces the expression levels of Cox-1 and Cox-2, subunits of respiratory complex IV translated by mitoribosomes, without changing the expression of COX-4 that is translated by cytosolic ribosomes (129, 152). As an evidence that tigecycline targets mitoribosomes, knockdown of the mitochondrial elongation factor EF-Tu mimics the effects of tigecycline (129, 153). Leukemia cells are particularly sensitive to tigecycline, being them heavily reliant on OXPHOS (174). Accordingly, experimental evidence in different human cancer cell lines support the idea that tigecycline exerts pro-apoptotic effects are more common in systems with high mitochondrial biogenesis and upregulated oxidative metabolism (130, 147–149, 151–153). Finally, tigecycline has been shown to have a synergistic effect with several chemotherapeutic drugs such as cisplatin (149, 152), paclitaxel (153), venetoclax (150), doxorubicin, vincristine (151), BRAF and MEK inhibitors (175).

#### 8.1.2 Actinonin

Actinonin is a peptidomimetic antibiotic naturally produced by actinomyces that arrests bacterial growth by inhibiting the peptide deformylase also identified in human cells (155, 176). Human mitochondrial peptide deformylase (HsPDF) is a metalloprotease that catalyzes the co-translational removal of the formyl group from N-terminal methionine of newly synthesized proteins (155). Expression of HsPDF was found significantly increased in breast, colon, and lung cancer tissues, suggesting that this enzyme may act as an oncogene to promote cancer cell proliferation (177). Inhibition of HsPDF by actinonin-based antibiotics reduces mitochondrial translation and OXPHOS complex assembly and inhibits the proliferation of several human cancer cell lines (129, 155, 178). The antiproliferative effects of actinonin has also been attributed to the activation of a mitoribosome quality control pathway that precedes the loss of mitochondrial respiration, although the molecular mechanisms are yet to be elucidated (154, 179). According to this interpretation, actinonin blocks the mitoribosomal polypeptyde exit tunnel, probably by trapping HsPDF on the LSU, leading to the accumulation of polypeptydyl-tRNAs in the P-site, which causes mitoribosome stall (179). Stalled mitoribosomes trigger a retrograde signal to the nucleus, that causes cell proliferation arrest (179). Sustained retrograde signal mediated by actinonin induces a mitochondrial decay pathway with degradation of mt-rRNAs, mt-mRNAs and mitoribosomes, which impairs the respiratory chain function (179). This mechanism of action may explain the anticancer effect of mitochondrial translation inhibitors even in cancer cells that do not rely on the OXPHOS for their energy demand.

### 8.2 Targeting Mitochondrial Protein Import

Carla Koehler’s research group has identified and characterized small molecules, including MitoBloCK-6 (MB-6) and MitobloCK-10 (MB-10), which interfere with mitochondrial protein import process in both yeast and mammalian cells. These two compounds attenuate precursor translocation by targeting different components of the import machineries: MB-10 binds Tim44, a component of the PAM complex, impairing the protein import into the matrix *via* TIM23 complex. On the other hand, MB-6 inhibits the sulfhydryl oxidase Erv1 and, in turn, Mia40 function in the import of intermembrane space proteins (180, 181). Interestingly, the human homolog of Erv1 (ALR) is found upregulated in hepatocellular carcinoma cell lines and tissues, while its silencing or inhibition through MB-6 impairs mitochondrial function and inhibits the proliferation of liver cancer cells (157, 182). MitoBloCKs can be a useful tool to study the role of mitochondrial translocation machinery in cancer, and the transcriptional and proteomic responses induced by accumulation of precursors in the cytosol, following the inhibition of protein import.

### 8.3 Targeting Mitochondrial Proteostasis

#### 8.3.1 Caseinolytic Protease P Modulators

Mitochondrial protease ClpP was found overexpressed in a subset of hematological and solid tumors where it is necessary for cancer cell viability (183–185). Inhibition of ClpP has been proposed as a strategy to impair OXPHOS and induce apoptosis in leukemic cells characterized by a high reliance on mitochondrial respiration (183). Bacterial ClpP inhibitors, ß-lactones derivatives (A2-32-01) and phenyl ester compounds (TG42, TG53), cross-react with human ClpP and show anti-proliferative and pro-apoptotic effects in human cancer cell lines (183, 186). However, these drugs are mainly a chemical tool to be used for functional studies and, therefore, further efforts are needed to design more specific drugs for human ClpP and reduce off-target effects. As for the inhibition, hyperactivation of ClpP also impairs OXPHOS and induces cancer cell death by uncontrolled degradation of ClpP respiratory chain substrates (187). Indeed, the ClpP activator imipridone ONC201 has shown efficacy as a single agent or in combination with other anti-cancer therapies in several solid and hematologic tumors and it is currently being tested in clinical trials (188).

#### 8.3.2 Lon Inhibitors

The matrix protease LONP1 is upregulated in different tumor types, including lymphomas, cervical, colorectal, bladder and non small cell lung cancer (189). Inhibition of LON by synthetic triterpenoids, 2-cyano-3, 12-dioxooleana-1,9(11)-dien-28-oic acid (CDDO) and its C-28 methyl ester derivative (CDDO-Me), show a cytotoxic effect in human cancer cell lines by impairing mitochondrial functions (190). However, these compounds exert inhibitory effects on different oncogenic factors, such as IκB kinase (IKK), ubiquitin-specific-processing protease 7 (USP7), erythroblastic oncogene B2 (ErbB2) or peroxisome proliferator activated receptor (PPAR)-γ, making the contribution of LONP1 inhibition to the anticancer effect unclear (161).

#### 8.3.3 TRAP1 Inhibitors

The molecular chaperone TRAP1 has been found upregulated in several cancer types, including breast, lung, prostate and colorectal cancers, where it is related to poor prognosis and advanced stages, whereas its genetic silencing induces an attenuation of cancer cells proliferation and *in vivo* tumor growth, providing a strong rationale for TRAP1 targeting as anticancer therapy (191).

Gamitrinibs are the first class of Hsp90 inhibitors that selectively accumulate in the mitochondrial matrix (162). Structurally, gamitrinibs are constituted by a backbone derived from 17-(allylamino)-17-demethoxygeldanamycin (17-AAG), needed to inhibit the ATPase activity of Hsp90, a linker region and a mitochondrial targeting module provided by one to four tandem repeats of cyclic guanidinium (gamitrinib-(G1-G4) or a triphenylphosphonium (gamitrinib-TPP) (162). Once in the mitochondrial matrix, gamitrinib inhibits the ATPase activity of mitochondrial HSP90 and TRAP1, inducing the accumulation of unfolded proteins with the consequent activation of the mtUPR and organelle disfunction (100, 117). Gamitrinib has been shown a “mitochondriotoxic” effect and anticancer activity in several human cancer cell lines, including squamous cell, breast, lung, prostate carcinoma and leukemia cells, and in *in vivo* models (162) in addition, it is recently approved to begin phase I clinical trial for advanced solid cancers.

Over the years, several efforts have been directed to designing others more selective TRAP1-targeting drugs with no effects on HSP90 activity, in order to reduce the overall cell toxicity. Rodanin et al. identified a strategy to selectively target TRAP1 ATPase domain by binding cationic appendages to the HSP90 inhibitors core (192). An alternative approach to achieve specific inhibition of TRAP1 is the identification of allosteric ligands disturbing the substructure that controls ATP hydrolysis by binding an allosteric site distal from the ATPase site (193). Highly selective small molecules targeting TRAP1 are needed to dissect the dynamics of client interaction under different conditions and thus the biochemical functions of this chaperone in cancer cells.

## 9 Conclusions

**Figure 3:**
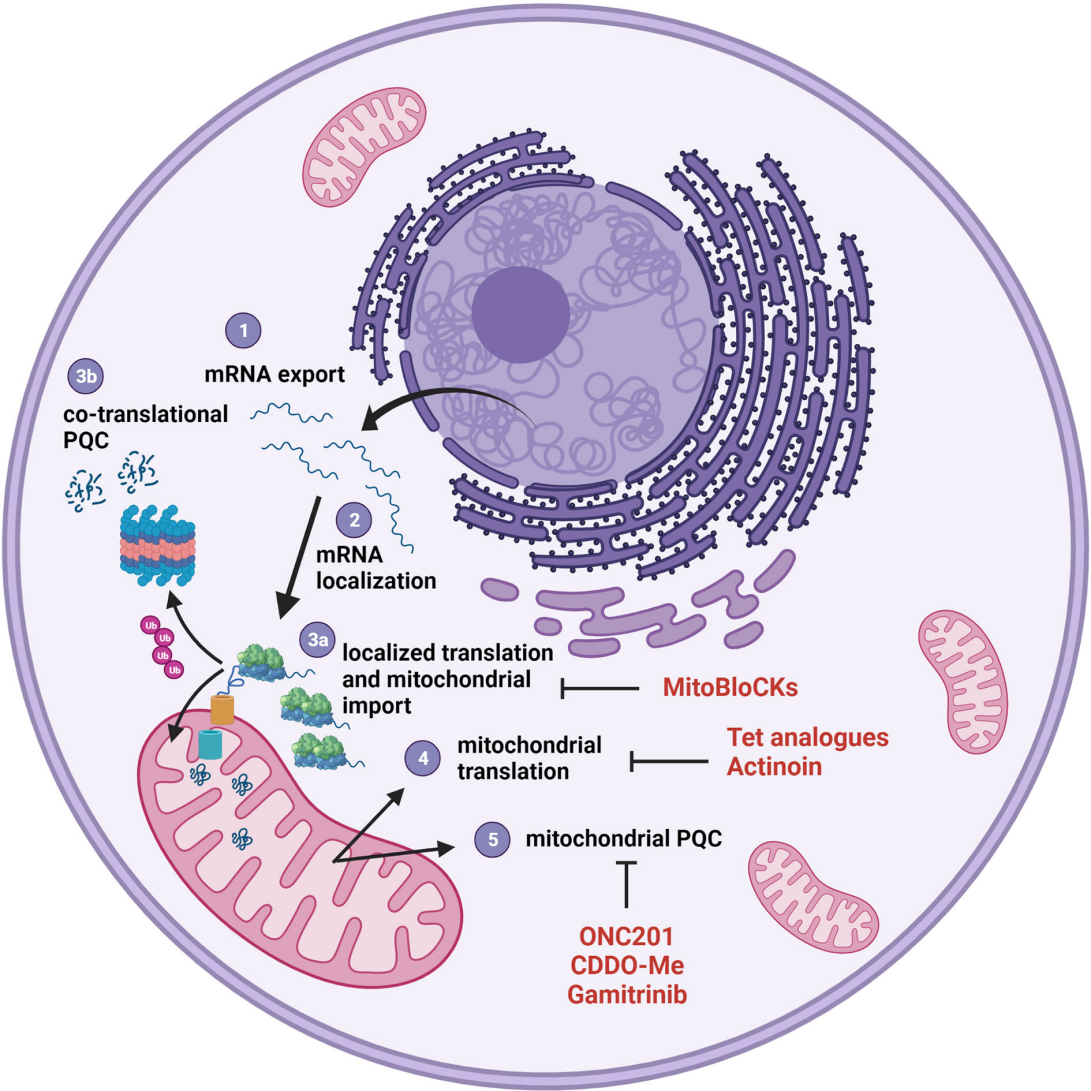
The mitochondrial proteome is controlled at several levels. The vast majority of mitochondrial proteins is encoded by the nuclear genome; therefore, the transcribed mRNAs must be exported from the nucleus (1) to be translated into the cytosol. This translation process can be compartmentalized through a localization of transcript to the organelle (2) with the contribution of the protein synthesis machinery. Proteins synthesized on the surface of mitochondria can be imported *via* TOM/TIM (3a) or discarded and degraded if they don’t pass the PQC step (3b). mRNA translation (4) and associated PQC (5) also occur in the mitochondrial matrix. All these steps can be potentially targeted with compound listed in **Table 1** and represented here in red.

Metabolic reprogramming is now recognized as one of the hallmarks of cancer (194). In recent years, an impressive amount of work has been done to identify energetic pathways that could be targeted for therapy, alone or in combination with more traditional anticancer drugs. At the same time, targeting biosynthetic pathways, as protein, nucleotide and lipid biosynthesis, have also attract attention and proposed the adoption of new drugs (or the repurposing of old ones), that have sometimes entered clinical trials (195, 196). Targeting protein synthesis seems an obvious strategy, considering that the aberrantly increased protein synthesis is one of the most common features of cancer cells, and that dysregulated ribosome biogenesis has been one of the first characteristics to be identified, in the form of hypertrophic nucleoli (197). Although the modulation of gene expression has been traditionally attributed to transcription regulation, in the last few years it has become increasingly evident that many processes were regulated at translational level (198), and among those, assembly and activity of respiratory complexes, whose subunits are inserted into the nascent macromolecular units in a co-translational manner (75). As an additional level of complexity, the respiratory complexes have a dual genetic origin, and are therefore composed both by proteins synthesized into the cytosol and later imported into the organelle and proteins synthesized in the matrix by the mitochondrial translational apparatus. Brilliant research projects mainly performed in yeast have shown in recent years that the two processes are tightly connected, co-regulated and coordinated, to ensure fine-tuned responses to energetic demands and nutrient availability (120). The available evidence shows that many of these processes (schematically summarized in **Figure 3**) are conserved in humans, and that in human cells a large amount of mRNAs encoding mitochondrial proteins can be found in the proximity of mitochondria (66). These transcripts can be locally translated, and the nascent protein co-translationally targeted to the organelle through a protein import channel (75). This complex presents, in turn, multiple connection with the respiratory chain, where mitochondrial-encoded subunits are simultaneously inserted co-translationally, based on the availability of the nuclear-encoded subunits (127). Given the central role played by these phenomena in cancer cells, we believe that shedding light on their regulation in cancer could provide an entire new avenue of both knowledge and therapeutic opportunities. Indeed, preliminary data suggest that cancer cells are particularly sensitive to translation uncoupling, and many compounds are already available to be tested in pre-clinical models. We believe that this scenario holds great promises either in terms of research advances and of opportunities for translation to the clinic.

## Author Contributions

DC, RA, DM, and FE wrote the manuscript. FE critically revised the manuscript. All authors contributed to the article and approved the submitted version.

## Funding

This work was supported by POR CAMPANIA FESR 2014/2020 [project “SATIN” (Sviluppo di Approcci Terapeutici INnovativi per patologie neoplastiche resistenti ai trattamenti)] and FRA (Finanziamento della Ricerca in Ateneo) 2020 grant to DM.

## Conflict of Interest

The authors declare that the research was conducted in the absence of any commercial or financial relationships that could be construed as a potential conflict of interest.

## Publisher’s Note

All claims expressed in this article are solely those of the authors and do not necessarily represent those of their affiliated organizations, or those of the publisher, the editors and the reviewers. Any product that may be evaluated in this article, or claim that may be made by its manufacturer, is not guaranteed or endorsed by the publisher.
